# Sonderlagen und Gefahrenabwehr in deutschen Krankenhäusern – eine Umfrage zum Ist-Zustand

**DOI:** 10.1007/s00101-023-01349-2

**Published:** 2023-10-19

**Authors:** M. von der Forst, E. Popp, M. A. Weigand, C. Neuhaus

**Affiliations:** Klinik für Anästhesiologie, Universität Heidelberg, Medizinische Fakultät Heidelberg, Im Neuenheimer Feld 420, 69120 Heidelberg, Deutschland

**Keywords:** Krankenhausalarm- und -einsatzplanung, MANV, Katastrophenschutz, Gefahrenabwehr, Sonderlagen, Katastrophenplanung, LEBEL (Lebensbedrohliche Einsatzlage), Emergency preparedness, Mass casualty incidents, Disaster planning, Hazard control, Special emergency situations

## Abstract

**Hintergrund und Fragestellung:**

Bei Ereignissen wie einem Cyberangriff oder einem Massenanfall von Verletzten müssen in Krankenhäusern Ad-hoc-Maßnahmen ergriffen werden. Die beteiligten Prozesse und Instrumente zur Gefahrenabwehr werden in der Krankenhausalarm- und -einsatzplanung (KAEP) festgelegt. Mit welchen Ressourcen und auf welche Sonderlagen sich Krankenhäuser vorbereiten, soll die vorliegende Studie erläutern.

**Methoden:**

Es wurde eine prospektive, explorative, anonyme Umfrage an Krankenhäusern in Deutschland durchgeführt. Eingeschlossen wurden Krankenhäuser, die sowohl über eine Innere Medizin als auch eine Chirurgie verfügen. Verantwortliche für Qualitäts‑/Risikomanagement wurden anhand eines standardisierten Fragebogens zu Ressourcen, Risiken und Inhalten der eigenen KAEP befragt.

**Ergebnisse:**

Es nahmen 95 Kliniken an der Umfrage teil, von diesen gaben 98 % (*n* = 93) an, über eine KAEP zu verfügen. Die Vorbereitung auf einzelne Szenarien war sehr unterschiedlich. In 60 % (*n* = 56) der befragten Kliniken existierte eine ärztlich besetzte Stabsstelle Krisen/Katastrophenmanagement. Eine Freistellung erfolgte in 12 Kliniken (ausnahmslos Schwerpunkt- oder Maximalversorger).

**Diskussion:**

Die meisten teilnehmenden Kliniken sind sich der Notwendigkeit einer KAEP bewusst und halten szenariospezifische Pläne vor. Lücken scheint es neben chemischen, biologischen und radionuklearen Lagen jedoch insbesondere im Bereich von Extremwetterereignissen und Infrastrukturausfällen zu geben. Es bedarf in Zukunft v. a. einer adäquaten Freistellung von ärztlichem Personal für den Bereich KAEP und einer Refinanzierung dieser Maßnahmen bei den Krankenhäusern.

**Zusatzmaterial online:**

Die Online-Version dieses Beitrags (10.1007/s00101-023-01349-2) enthält den zugrunde liegenden Fragebogen.

## Abstract (en)

**Background and research question:**

In case of events such as a cyber attack or a mass casualty incident, ad hoc measures have to be taken in hospitals. As part of the critical infrastructure, hospitals are required by law to prepare, update and exercise alarm and emergency plans for various special situations. The processes and instruments involved for emergency response are defined in the hospital alert and emergency planning. The present study aims to explain with which resources and for which special situations hospitals are prepared.

**Methods:**

A prospective, exploratory, anonymous survey of hospitals in Germany was conducted. Hospitals with both internal medicine and surgery departments were included. Out of 2497 hospitals listed in the German Hospital Directory (www.deutsches-krankenhaus-verzeichnis.de), 1049 met the inclusion criteria. After correcting for hospital groups with shared administrations, 850 employees were identified and contacted by e‑mail. Quality and risk management managers were asked about resources, risks, and content of their own hospital alert and emergency planning using a standardized questionnaire. The survey was conducted using the online platform EFS Survey (Tivian XI GmbH, Cologne) via www.unipark.de. Access to the survey was via a nonpersonalized hyperlink. Apart from the size and type of hospital surveyed, no data were collected that would allow identification of an individual person.

**Results:**

Of the participating hospitals 45% (*n* = 43) were primary care hospitals, 24% (*n* = 23) were specialty care hospitals, 10% (*n* = 9) were nonuniversity maximum care hospitals, and 21% (*n* = 20) were university maximum care hospitals. In total 95 hospitals participated in the survey, of which 98% (*n* = 93) reported having a hospital alert and emergency plan. Preparation for individual scenarios varied widely. Of the participating hospitals 45% (*n* = 43) reported having been the target of cyber attacks with an emphasis on maximum care hospitals (55%, *n* = 11 of 20). Technical redundancy for computer systems is available in 67% (*n* = 63) of participating hospitals, while independent means of communication exist in 50% (*n* = 47) of hospitals. A physician-staffed crisis and disaster management unit existed in 60% (*n* = 56) of the surveyed hospitals. At least a part time position for planning issues was installed in 12 hospitals.

**Conclusion:**

Most participating hospitals are aware of the need for a hospital alert and emergency plan and have various scenario-specific plans in place. Especially mass casualty events, fire and hospital evacuation scenarios are uniformly covered among participating hospitals; however, gaps appear to exist not only for chemical, biological or radionuclear situations but also especially in the area of extreme weather events and infrastructure failures. Only about two thirds of all participating hospitals have contingency plans for water supply and/or heating failures. An important limitation of the study is the comparatively low response rate of 12.9% (*n* = 95 of 850). While primary care hospitals were underrepresented in the study, 32% of Germany’s larger hospitals (> 800 beds) participated. In the future, there is a particular need to engage enough medical staff in the area of hospital alert and emergency planning and refunding of these measures by hospitals.

## Hinführung zum Thema

Zum Schutz vor Gefahren haben u. a. das Bundesamt für Bevölkerungsschutz und Katastrophenhilfe (BBK) sowie die Deutsche Arbeitsgemeinschaft Krankenhaus-Einsatzplanung (DAKEP) e. V. Empfehlungen für die Erstellung der Krankenhausalarm- und -einsatzplanung verfasst [3]. Inwiefern die sich verändernden Risiken für Krankenhäuser und das Bewusstsein für Lieferengpässe oder Ausfälle der eigenen Infrastruktur nach der Coronapandemie bereits Eingang in die Praxis gefunden haben, und wo aus Sicht der Kliniken bei diesem Thema weiterer Handlungsbedarf besteht, soll in der vorliegenden Arbeit evaluiert werden.

## Hintergrund

Die letzten Jahre haben gezeigt, dass die „klassischen“ Bedrohungen für Krankenhäuser, z. B. ein Brand oder der Massenanfall von Verletzten (MANV), in der Planung durch zwei wesentliche Kategorien ergänzt werden sollten. Dies sind einerseits Lagen mit einem kriminellen Hintergrund (z. B. Cyberangriffe, Amok, Sabotage) und andererseits Infrastrukturausfälle (z. B. Strom, Wasser, Kommunikation) [8, 9, 11, 17, 27]. Nach dem „konsequenzbasierten Modell“ von Wurmb et al. führen diese Szenarien primär regelhaft zu einer Störung der Funktionalität und sekundär zu einer Einschränkung der Behandlungskapazität [25, 29].

Krankenhäuser sind als Teil der kritischen Infrastruktur durch länderspezifische Vorgaben gesetzlich dazu verpflichtet, Alarm- und Einsatzpläne für verschiedene Sonderlagen aufzustellen, fortzuschreiben und zu beüben [2, 3]. Generell sollen, basierend auf einer individuellen Risikoanalyse des jeweiligen Krankenhauses, die Identifikation und Priorisierung der potenziellen Gefährdungsszenarien erfolgen. Die Prozesse und Maßnahmen zur Gefahrenabwehr werden als Krankenhausalarm- und -einsatzplanung (KAEP) bezeichnet. Die übergeordneten Schutzziele der KAEP und der ausführenden Krankenhauseinsatzleitung (KEL) sind in der Regel die Gewährleistung der Sicherheit von Personal und Patienten sowie konsekutiv die Aufrechterhaltung der Funktionalität des Krankenhauses [3, 4].

## Onlinebefragung

Das Ziel der durchgeführten Studie war eine Analyse des Ist-Zustands in Bezug auf vorbereitende Maßnahmen zur Gefahrenabwehr in Krankenhäusern. Hierfür wurde eine prospektive, explorative, anonyme Umfrage an Krankenhäuser in Deutschland verschickt. Dabei wurden für den Bereich Qualitätsmanagement/Risikomanagement verantwortliche Personen deutscher Krankenhäuser über die zur Verfügung stehenden Ressourcen, die Inhalte und den Stand der eigenen KAEP befragt. Die Mitarbeitenden wurden über das Deutsche Krankenhaus Verzeichnis (www.deutsches-krankenhaus-verzeichnis.de) identifiziert und per E‑Mail kontaktiert. In die Auswahl wurden Krankenhäuser eingeschlossen, die sowohl über eine Innere Medizin als auch eine Chirurgie verfügen und somit mutmaßlich an der Regelversorgung teilnehmen. Die erste Einladung zur Teilnahme an der Umfrage erfolgte am 07.08.2022, eine Erinnerungsmail wurde am 22.08.2022 versendet. Die Umfrage wurde nach einer Laufzeit von 5 Wochen am 09.09.2022 beendet.

Die Umfrage erfolgte mit der Online-Plattform EFS Survey (Fa. Tivian XI GmbH, Köln) über www.unipark.de. Der Zugang zur Umfrage erfolgte über einen nichtpersonalisierten Hyperlink. Neben Größe und Art der befragten Klinik wurden keine Daten erhoben, die die Identifikation einer Einzelperson ermöglichen (wie z. B. Mail-Adresse, Arbeitsplatz etc.). Der Zeitaufwand für den Fragebogen war mit ca. 10–15 min angesetzt.

Die erhobenen Daten wurden anhand von deskriptiver Statistik analysiert. Diese erfolgte unter Angabe von absoluten und relativen Häufigkeiten bzw. deren Mittelwert und Standardabweichung. Kontinuierliche Merkmale wurden mittels *t*-Test und kategoriale Daten mittels Chi^2^-Test verglichen. Das Signifikanzniveau wurde auf 5 % festgelegt. Die als Ergebnisse gewonnenen *p*-Werte besitzen einen rein deskriptiven Charakter; fehlende Werte wurden nicht imputiert. Die Analysen wurden mit Microsoft Excel (Version 16.63.1, Fa. Microsoft Corp., Redmond, WA, USA) und EFS Reporting+ (Fa. Tivian XI GmbH, Köln) durchgeführt.

Die Erhebung wurde in Übereinstimmung mit der Deklaration von Helsinki in der aktuellen Fassung durchgeführt. Zu Beginn der Umfrage erschien der Hinweis, dass sämtliche erhobenen Daten anonym gespeichert werden und ein Abbruch der Befragung jederzeit möglich ist. Es erfolgte keine gesonderte Probandenaufklärung. Die Teilnehmer hatten weder ein persönliches Risiko noch einen Nutzen von ihrer Teilnahme, es wurde jedoch ein wissenschaftlicher Erkenntnisgewinn der Gesamtstudie erwartet.

Das Studienprotokoll wurde vor Studienbeginn durch die Ethikkommission der Medizinischen Fakultät Heidelberg positiv begutachtet (S-468/2022). Die Studie erfüllt die Anforderungen des STROBE-Statements [26].

## Ergebnisse

**Figure 1:**
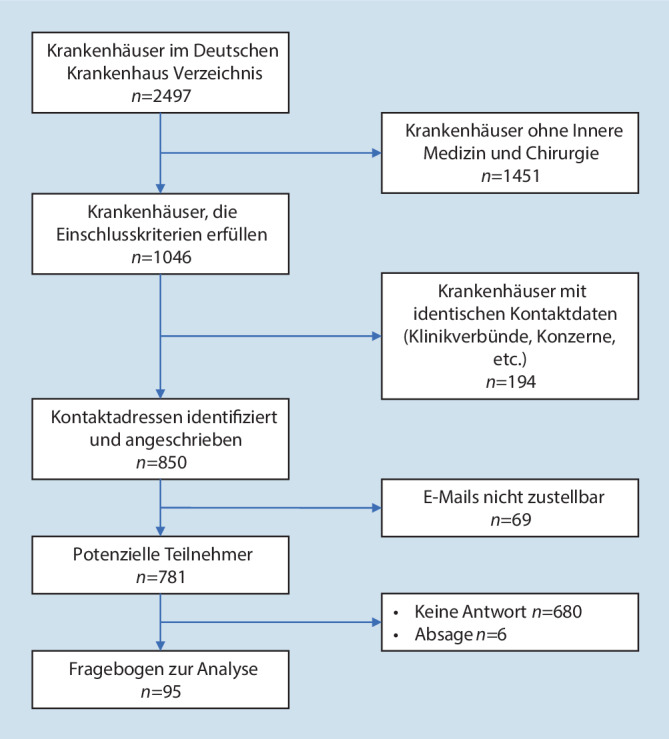

Das Deutsche Krankenhaus Verzeichnis listete zum Zeitpunkt der Umfrage 2497 Krankenhäuser. Nach Abzug von Fachkrankenhäusern und Rehabilitationseinrichtungen wurden 1049 Kliniken identifiziert, die die Einschlusskriterien erfüllten. Nach Abzug von Mehrfachnennungen (teilweise waren Mitarbeitende in Klinikverbünden für mehrere Kliniken zuständig) wurden 850 E‑Mail-Adressen kontaktiert. Hiervon nahmen im Zeitraum vom 07.08.2022–09.09.2022 95 Kliniken an der Umfrage teil. Sechs Kliniken beantworteten die Einladungsmail mit einer Absage, und 69 E-Mails konnten nicht zugestellt werden (Abb. 1).

Die mittlere Bearbeitungszeit lag bei 10 min 58 s. Es muss davon ausgegangen werden, dass die Umfrage zur Vorbereitung der Eingaben teilweise mehrfach aufgerufen wurde; dies ergibt sich aus der Diskrepanz zwischen der Anzahl aufgerufener (*n* = 303) und abgeschlossener (*n* = 95) Fragebogen.

### Struktur der teilnehmenden Krankenhäuser und deren Krankenhauseinsatzleitung

**Figure 2:**
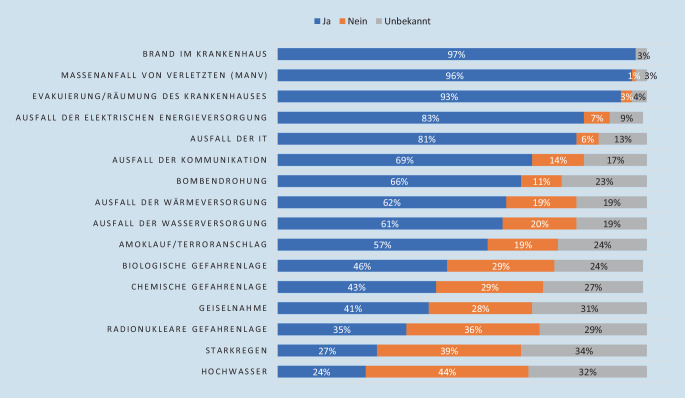

Die Gesamtbeteiligung an der Umfrage betrug 12,9 %. Von den teilnehmenden Kliniken waren 45 % (*n* = 43) Häuser der Grund- und Regelversorgung, 24 % (*n* = 23) Häuser der Schwerpunktversorgung, 10 % (*n* = 9) Häuser der nichtuniversitären Maximalversorgung und 21 % (*n* = 20) Häuser der universitären Maximalversorgung (Tab. 1). Von den an der Umfrage teilnehmenden Kliniken gaben 98 % (*n* = 93) an, über eine KAEP zu verfügen. Die Klinikeinsatzleitung nutzte in 94 % (*n* = 89) eine Stabsstruktur. Bei 95 % (*n* = 90) der teilnehmenden Kliniken werden ereignisspezifische Einsatzpläne/Verfahrensanweisungen vorgehalten (Abb. 2).

**Table 1:** 

Bettenzahl	Median	Min	Max
Grund- und Regelversorger (*n* = 43)	278	97	455
Schwerpunktversorger (*n* = 23)	600	468	825
Maximalversorger (*n* = 29)	1423	836	2300

### Cyberangriffe und technische Redundanz

Rund die Hälfte der teilnehmenden Kliniken (45%, *n* = 43) gaben an, bereits Ziel von Hackerangriffen bzw. Cyberattacken gewesen zu sein, wobei hiervon vermehrt die universitären Maximalversorger betroffen waren (55 %, *n* = 11 von 20). Dementsprechend wurde von 55 % (*n* = 52) der teilnehmenden Kliniken die Sicherheit von Patienten- oder Forschungsdaten als relevantes Problem eingeschätzt; auf universitäre Maximalversorger traf dies signifikant häufiger zu als auf Teilnehmer anderer Versorgungsstufen (*p* < 0,05). Zu Fragen der technischen Redundanz gaben 67 % (*n* = 63) der teilnehmenden Kliniken an, EDV/IT-Strukturen vorzuhalten, die nicht im Routinebetrieb eingesetzt werden. Kommunikationstechnik wie z. B. Einsatzstellenfunk für den Krisenfall, wird von 50 % (*n* = 47) der teilnehmenden Kliniken vorgehalten. In 20 % (*n* = 19) bzw. 28 % (*n* = 26) der Umfragen konnte hierzu jedoch keine Aussage gemacht werden.

### Technische Systeme im Bereich Brandschutz und Fluchtwege

Im Bereich der technischen Maßnahmen für den Brandschutz gab die überwiegende Mehrzahl der Teilnehmer an, über Brandmeldeanlagen und Rauch- und Feuerschutztüren zu verfügen (97 %; *n* = 92); 3 Kliniken konnten hierzu keine Aussage machen. Rauchabzugsanlagen waren bei 86 % (*n* = 82) installiert, wobei 7 % (*n* = 7) nicht über derartige Systeme verfügen. Für die Notfallalarmierung und -lenkung verfügen 74 % (*n* = 70) der teilnehmenden Kliniken über Fluchtwegsicherungssysteme und 87 % (*n* = 83) über eine Notbeleuchtung. Eine Sprachalarmanlage für Alarmierungs- und Evakuierungsaufgaben wurde von 47 % (*n* = 45) der Teilnehmer als vorhanden angegeben; dies wurde von 37 % (*n* = 35) verneint. Notfall- und Gefahrenreaktionssysteme für die Alarmierung von z. B. Sicherheitsdiensten oder Polizei waren bei 52 % (*n* = 49) der teilnehmenden Kliniken vorhanden, weitere 27 % (*n* = 26) verneinten derartige Installationen.

### Personelle Aufstellung im Bereich KAEP

**Figure 3:**
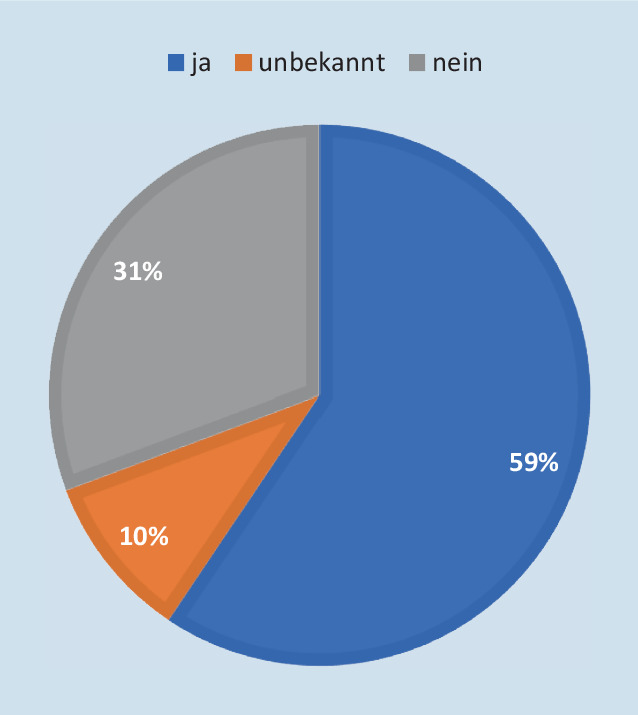

**Figure 4:**
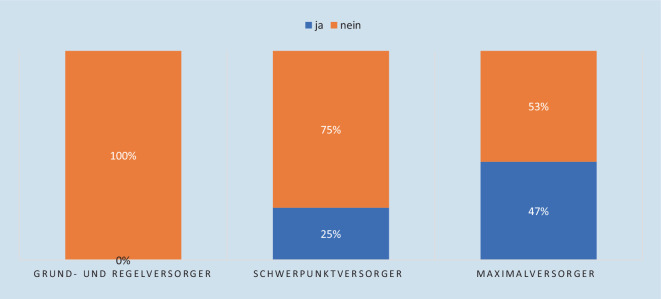

In 60 % (*n* = 56) der teilnehmenden Kliniken existiert eine ärztlich besetzte Stabsstelle Krisen/Katastrophenmanagement, während knapp ein Drittel der Kliniken nicht über eine solche Struktur verfügt (Abb. 3). Eine Freistellung hierfür erfolgt in 12 der teilnehmenden Kliniken (ausnahmslos Schwerpunkt- oder Maximalversorger, Abb. 4).

## Diskussion

### Kongruenz der aktuellen KAEP mit den veröffentlichten Empfehlungen

Durch die Gegebenheiten der letzten Jahre, wie die Coronapandemie, den Krieg in der Ukraine und die Zunahme von Extremwetterereignissen (z. B. die Flutkatastrophe im Ahrtal 2021), rückte das Thema KAEP vermehrt in den Fokus. Gleichzeitig wurde durch die Veröffentlichung des *Handbuch Krankenhausalarm- und -einsatzplanung* vom BBK und weiteren Fachgesellschaften im Jahr 2020 den Kliniken ein Rahmengerüst zur Durchführung der KAEP gegeben [3].

Die Ergebnisse der vorliegenden Umfrage zeigen eine Übersicht über die Kongruenz der durch die Krankenhäuser berücksichtigten Sonderlagen im Vergleich zu den Empfehlungen des BBK (Abb. 2). Hierbei wird deutlich, dass chemische, biologische und radionukleare Lagen (CBRN) sowie Extremwetterereignisse im Vergleich zu „klassischen“ Risiken wie Brand oder MANV unterrepräsentiert sind; weniger als die Hälfte der teilnehmenden Kliniken haben hierfür konkrete Handlungsanweisungen. CBRN-Lagen haben als Gemeinsamkeit häufig die Notwendigkeit einer Dekontamination und sind spätestens seit dem Saringasanschlag in Tokio 1995 in den Köpfen präsent, werden aber, mutmaßlich aufgrund der hohen Komplexität, oft paradoxerweise nur unzureichend vorgeplant [19]. Interessant ist auch, dass nur 46 % der teilnehmenden Kliniken Handlungsanweisungen für eine biologische Lage vorhalten, wobei hierzu auch ein Pandemieplan zählt, dessen Notwendigkeit spätestens seit der Coronapandemie unbestritten sein dürfte.

Einsatzlagen, bei denen die eigene Versorgungsinfrastruktur betroffen ist, wie z. B. der Ausfall von Wasser- oder Wärmeversorgung, werden aktuell nur von knapp zwei Drittel der Kliniken explizit berücksichtigt; Extremwetterereignisse wie Starkregen und Hochwasser werden sogar nur in jeder vierten Klinik adressiert. Insbesondere Sonderlagen aus diesen Bereichen haben jedoch Krankenhäuser in der Vergangenheit vor ernsthafte Probleme gestellt [9, 12]. Neben direkter Effekte auf das Krankenhaus kommt es bei solchen Ereignissen häufig auch zu einer vermehrten Inanspruchnahme durch die Bevölkerung, z. B. aufgrund der Zerstörung der umliegenden Infrastruktur [23]. Es ist aufgrund der globalen Entwicklung hin zu extremeren Wetterbedingungen anzunehmen, dass solche Ereignisse eher zunehmen werden.

### IT-Sicherheit

Die Tatsache, dass über 80 % der teilnehmenden Kliniken über Pläne für einen IT-Ausfall verfügen, zeigt eine Zunahme im Vergleich zu einer Umfrage von 135 Kliniken in Baden-Württemberg 2019 [21]. Fallberichte aus den vergangenen Jahren dokumentieren, dass 47 % der Krankenhäuser im National Health Service in den Jahren 2015 und 2016 von Hackerangriffen betroffen waren [17]. Weitere Ausfälle und Beeinträchtigungen der Patientenversorgung durch Malware, u. a. in England, Deutschland und Australien, wurden ebenfalls berichtet [6]. Diese Zahlen decken sich mit den Ergebnissen der vorliegenden Umfrage, bei der 45 % der Kliniken angeben, bereits Hackerangriffen oder Cyberattacken ausgesetzt gewesen zu sein.

### MANV und Brand

Auch die Szenarien MANV, Brand und Evakuierung werden von nahezu allen teilnehmenden Krankenhäusern adressiert. Insbesondere in den Bereichen des baulichen Brandschutzes und Evakuierung mag dies möglicherweise an den gesetzlichen Vorgaben liegen [16]. Das Szenario MANV hingegen ist meist u. a. durch Ereignisse in der Vergangenheit, wie z. B. die Massenpanik bei der Loveparade in Duisburg (2010), im Fokus der Kliniken, eine Vorbereitung dieses Szenarios wird aber auch für die Zertifizierung als Traumazentrum gefordert und ist deshalb oft unabdingbar [8].

### Vorhaltung von personeller Expertise für die KAEP

Neben konkreten Plänen für einzelne Sonderlagen wird es, aufgrund der Vielzahl möglicher Bedrohungen, in Zukunft jedoch zunehmend an Bedeutung gewinnen, in den Krankenhäusern fortlaufend personelle Expertise für den Bereich KAEP vorzuhalten [29]. Die durch Fachleute im Ernstfall eingebrachte adaptive Kapazität kann dabei häufig von kritischer Bedeutung sein, da auch die besten theoretischen Planungen häufig Lücken aufweisen [15]. Zudem besteht nicht zwingend eine lineare Beziehung zwischen Vorhalteaufwand und Erhöhung der Sicherheit [14]. Die Erfahrungen zeigen, dass eine Komplexitätserhöhung des Gesamtsystems, z. B. durch zu detaillierte Pläne oder „verschachtelte“ Redundanzen dessen Leistungsfähigkeit paradox schwächen können, dieses Phänomen wird als „defense in depth fallacy“ bezeichnet [24]. Es erscheint deshalb sinnvoll, bereits im Routinebetrieb feste (Stabs‑)Stellen einzurichten, welche eine kontinuierliche Adaptation der KAEP sicherstellen und die Kernelemente Risikoanalyse, Alarmierung und Mitarbeiterfortbildung bearbeiten. In der Vergangenheit zeigte sich, dass nur 53 % der Mitarbeiter die KAEP in ihren Kliniken kannten und lediglich in einem Drittel der Krankenhäuser Übungen durchgeführt wurden; in neueren Umfragen ergab sich hier eine deutliche Besserung [10, 21].

In der vorliegenden Studie haben knapp zwei Drittel der teilnehmenden Kliniken eine solche ärztliche besetzte Stabsstelle bereits eingerichtet (Abb. 3). Für diese verantwortungsvolle Aufgabe wird eine „an die Größe des Krankenhauses angepasste“, anteilige Freistellung empfohlen, diese ist auch für die erfolgreiche Zertifizierung des KAEP nach KTQ Voraussetzung [3, 7]. An dieser Stelle zeigt sich, dass insbesondere in kleineren Kliniken ärztliches Personal diese Aufgaben „on top“ erledigen muss, keiner der 43 teilnehmenden Regelversorger und nur 25 % der 23 Schwerpunktversorger hatten hierfür eine personelle Freistellung. Auch bei Maximalversorgern wurde nur in etwa der Hälfte der Fälle ärztliches Personal für die Stabsstelle freigestellt. Diese Befunde decken sich mit den Ergebnissen einer Online-Befragung der DAKEP-Mitglieder, welche für Kliniken kleiner als 500 Betten ebenfalls kaum Personalfreistellungen ergab [13].

Einer der Gründe, der sich auch in den Freitextantworten widerspiegelt, sind der auf den Krankenhäusern lastende ökonomische Druck und die nichtvorhandene Refinanzierung von Maßnahmen zur KAEP. An dieser Stelle besteht dringender Handlungsbedarf, damit die Kliniken als Teil der kritischen Infrastruktur in die Lage versetzt werden, sich adäquat auf drohende Sonderlagen vorzubereiten, da eine fehlende Vorbereitung direkt mit den auftretenden Schäden assoziiert werden kann [20].

### Grenzen der vorliegenden Studie

Eine der Hauptlimitationen der vorliegenden Umfrage ist die im Vergleich zur Zahl der kontaktierten Kliniken geringe Rücklaufquote von 12,9 % (*n* = 95 von 850). Potenzielle Gründe hierfür sind einerseits die mit etwa 5 Wochen limitierte Dauer der Umfrage, andererseits der Zeitpunkt gegen Ende der Haupturlaubssaison. Zudem gab es keine monetären bzw. sonstigen Anreize für die Studienteilnehmer [5]. Es muss beachtet werden, dass, verglichen mit Strukturdaten des Statistischen Bundesamtes, Krankenhäuser der Grund- und Regelversorgung in der vorliegenden Arbeit unterrepräsentiert und Krankenhäuser der Maximalversorgung überrepräsentiert waren [25]. Es zeigt sich gleichzeitig ein möglicher „selection bias“, wenn man argumentiert, dass das Thema KAEP bei Kliniken des Maximalversorgung einen höheren Stellenwert genießt, wodurch auch das Antwortverhalten auf die Umfrage positiv beeinflusst wird. Dennoch liegt die Stärke der Befragung darin, dass durch gezielte Adressatenauswahl eine homogene Stichprobe mit für das Thema relevanten Teilnehmern entstanden ist [1, 22]. Durch die Anonymität der Online-Umfrage ist zudem davon auszugehen, dass soziale Erwünschtheit nur eine untergeordnete Rolle spielt und diese hierbei anderen Umfrageformaten überlegen ist [18]. Die Teilnahme von 30 Kliniken mit mehr als 800 Betten, dies entspricht 32 % der in Deutschland gemeldeten 95 Häuser dieser Größe, untermauert die Ergebnisse aus dem Bereich dieser Subgruppe [25].

Ein weiterer Aspekt sind die länderspezifischen Unterschiede für die Krankenhausalarm- und -einsatzplanung, mit teilweise sehr abstrakt ausformulierten Vorgaben. Es ist anzunehmen, dass aufgrund der unterschiedlichen Gesetzeslage einzelner Bundesländer der Vorbereitungsgrad der jeweiligen Krankenhäuser variiert. In der vorliegenden Umfrage wurde die Umsetzung der durch das BBK empfohlenen Maßnahmen unabhängig von der geltenden Gesetze abgefragt. Aufgrund der Anonymität der Umfrage ist eine weitere Untersuchung regionaler Unterschiede jedoch unmöglich.

### Zusammenfassung zum IST-Zustand der KAEP in Deutschland

Die vorliegende Umfrage liefert einen aktuellen Überblick zu Maßnahmen der KAEP bei Krankenhäusern unterschiedlicher Größe in der Bundesrepublik Deutschland. Die meisten Kliniken sind sich der Notwendigkeit eines KAEP bewusst und halten szenariospezifische Pläne vor. Lücken scheint es neben CBRN-Lagen insbesondere im Bereich von Extremwetterereignissen und Infrastrukturausfällen zu geben. Um diese insgesamt positive Entwicklung in Zukunft voranzutreiben, bedarf es v. a. einer adäquaten Freistellung von ärztlichem Personal z. B. in Form einer Stabsstelle zur Bewältigung von Aufgaben der KAEP und einer Refinanzierung dieser Maßnahmen bei den Krankenhäusern.

## Fazit für die Praxis

- Die Vorbereitungen der deutschen Krankenhäuser auf Sonderlagen decken sich an vielen Stellen mit den herausgegebenen Empfehlungen des Bundesamtes für Bevölkerungsschutz und Katastrophenhilfe.
- Trotz der gestiegenen Aufmerksamkeit für das Thema und der zunehmenden Vorbereitung der Krankenhäuser auf Cyberangriffe sollten insbesondere Gefahren wie z. B. Extremwetterereignisse und Ausfälle der eigenen Infrastruktur nicht aus dem Blick verloren werden.
- Es sollten in den Krankenhäusern Experten für die Erstellung der KAEP benannt und diesen ausreichende Ressourcen (u. a. Freistellung) zur Verfügung gestellt werden.
- Die Krankenhäuser sollten sich für eine Refinanzierung der durch die gesetzlichen Maßnahmen geforderten Vorbereitung auf Sonderlagen im Rahmen der Daseinsfürsorge einsetzen.

### Supplementary Information




