# Supplementary material for: Special emergency situations and hazard control in German hospitals—A survey on the current state
Source: Anaesthesiologie. 2023 Oct 19;72(11):784–90. [Article in German] doi: 10.1007/s00101-023-01349-2 (PMC10615912; doi:10.1007/s00101-023-01349-2)
Supplement: Supplementary file 1 [file 101_2023_1349_MOESM1_ESM.pdf]

Zusatzmaterial zum Beitrag „Sonderlagen und Gefahrenabwehr in deutschen Krankenhäusern – Eine Umfrage zum Ist-Zustand“ von M. von der Forst, E. Popp E, M.A. Weigand, Ch. Neuhaus (2023) in *Die Anaesthesiologie*.

Beitrag und Zusatzmaterial stehen Ihnen auf www.springermedizin.de zur Verfügung. Bitte geben Sie dort den Beitragstitel in die Suche ein.

Fragebogen

1 Startseite

Sehr geehrte Teilnehmerin, sehr geehrter Teilnehmer,

wir führen eine anonyme Umfrage zum Thema „**Maßnahmen für die Sicherheit in Krankenhäusern**“ durch. Hierfür würden wir Ihnen gerne einige Fragen zu den technischen, organisatorischen und personellen Maßnahmen in Ihrem Krankenhaus stellen, die zur Risikominimierung und Gefahrenabwehr eingesetzt werden. Die Beantwortung wird ca. 15 Minuten Zeit in Anspruch nehmen.

Wir erfassen dabei **keine** personenbezogenen Daten, Ihre Teilnahme ist **komplett anonym**. Die Teilnahme an der Studie erfolgt freiwillig. Falls Sie teilnehmen möchten, bitten wir Sie, die folgende Umfrage bis zu Ende zu beantworten; **ein Abbruch ist jederzeit möglich**. Inkomplett ausgefüllte Fragebögen werden nicht ausgewertet, erst mit der vollständigen Bearbeitung des Fragebogens erklären Sie Ihre Einwilligung. Die erhobenen Daten werden ausschließlich zu Zwecken dieser Studie verwendet. Die Studienleitung wird alle angemessenen Schritte unternehmen, um den Schutz der Daten gemäß den Datenschutzstandards der Europäischen Union zu gewährleisten. Die Daten sind gegen unbefugten Zugriff gesichert und werden für 5 Jahre aufgehoben. Ihnen entstehen aus der Teilnahme weder persönlicher Nutzen noch Risiken.

Die Umfrage wurde der Ethikkommission der Medizinischen Fakultät der Universität Heidelberg vorgelegt und erhielt ein positives Votum (Aktenzeichen S-468/2022 vom 03.08.2022).

Für Rückfragen erreichen Sie uns jederzeit unter patientensicherheit.anae@med.uni-heidelberg.de

2 Krankenhausinfo

Welcher Versorgungsstufe gehört Ihr Krankenhaus an?

Bitte wählen Sie eine Option

- ○ Grund- und Regelversorgung
- ○ Schwerpunktversorgung
- ○ Maximalversorgung (nichtuniversitär)
- ○ Maximalversorgung (universitär)

Über wieviele Betten verfügt Ihr Krankenhaus?

Bitte geben sie einen ganzen Wert ein

3 Kriminaldelikte

Kriminaldelikte wie z.B. Diebstahl, Raub, Vandalismus/Sachbeschädigung etc. stellen ein Problem dar

Bitte wählen Sie eine Antwortmöglichkeit

- ○ ja
- ○ nein

Folgende Sachwerte waren in unserem Krankenhaus bereits Ziel krimineller Handlungen:

Bitte bewerten Sie alle Kategorien

	Ja	Nein	Unbekannt
Medikamente	○	○	○
Gerätschaften	○	○	○
Gebäude	○	○	○
Einrichtungsgegenstände	○	○	○
Wertsachen	○	○	○

IT-Sicherheit von Patienten- oder Forschungsdaten stellt ein relevantes Problem dar

Bitte wählen Sie eine Antwortmöglichkeit

- ○ ja
- ○ nein

Unser Krankenhaus war bereits Ziel folgender krimineller Handlungen im IT-Bereich:

Bitte bewerten Sie alle Kategorien

	Ja	Nein	Unbekannt
Hackerangriff/Cyberattacken	○	○	○
Raub von Patientendaten	○	○	○
Raub von Forschungsdaten	○	○	○

4 Technische Ausstattung

Das Krankenhaus verfügt über:

Bitte bewerten Sie die folgenden Aussagen

	ja	nein	unbekannt
**Bereich Feuerschutz**			
Brandmeldeanlage	○	○	○
Rauch- und Feuerschutztüren	○	○	○
Rauchabzugsanlagen	○	○	○
**Notfallalarmierung- und lenkung**			
Fluchtwegsicherungssysteme (sichern gegen unkontrollierten Ein- bzw. Austritt von Personen, und sorgen dafür, dass Rettungswegetüren im Gefahrfall freigeschaltet und geöffnet werden)	○	○	○
Notbeleuchtung	○	○	○
Sprachalarmanlage (Beschallungsanlage, die aufgrund ihres besonderen Aufbaus und ihrer hohen Sprachverständlichkeit für Alarmierungs- und Evakuierungsaufgaben geeignet ist)	○	○	○
Einbruchmeldeanlagen (Alarmanlage z.B. mit Glasbruch- /Bewegungssensoren)	○	○	○
Notfall- und Gefahrenreaktions-System (NRGS, "Hausalarm" zur Alarmierung von z.B.Sicherheitsdienst oder Polizei)	○	○	○
**Sonstiges**			
Rohrpostanlagen	○	○	○

Das Krankenhaus nutzt Videoüberwachung:

Bitte bewerten Sie die folgenden Aussagen

	ja	nein	unbekannt
Im öffentlichen Bereich (z.B. Eingänge, Anmeldung, Ambulanzen, Wartebereiche, Kantine)	○	○	○
Im nichtöffentlichen Bereich (z.B. Patientenzimmer, Behandlungsräume, Funktions-/ Lager- /Technikräume, Labor- /OP-Bereich)	○	○	○
Zur Überwachung der Freigelände (Videoüberwachung der Aussenanlage, Parkplätze etc)	○	○	○

Das Krankenhaus nutzt folgende Möglichkeiten der Zugangssteuerung:

Bitte bewerten Sie die folgenden Aussagen

	ja	nein	unbekannt
Mechanisch (Schlüssel)	○	○	○
Elektronisch (Chipkarte/Mitarbeiterausweis/Dongle)	○	○	○
Biometrisch (Fingerabdruck)	○	○	○

Es wird IT/EDV-Infrastruktur als Backup vorgehalten, die nicht im Routinebetrieb eingesetzt wird

Bitte wählen Sie eine Antwortmöglichkeit

- ○ ja
- ○ nein
- ○ unbekannt

Es wird Kommunikationstechnik als Backup vorgehalten, die nicht im Routinebetrieb eingesetzt wird (Einsatzstellenfunk)

Bitte wählen Sie eine Antwortmöglichkeit

- ○ ja
- ○ nein
- ○ unbekannt

5 Organisatorische Maßnahmen

Das Krankenhaus verfügt über:

Bitte bewerten Sie die Antwortmöglichkeiten

	Ja	Nein	Unbekannt
Krankenhausalarm- und Einsatzplan (KAEP)	○	○	○
Klinikeinsatzleitung (KEL) mit Stabsstruktur	○	○	○
Ereignisspezifische Einsatzpläne/Verfahrensanweisungen/Checklisten	○	○	○
Hausordnung	○	○	○

Das Krankenhaus verfügt über ereignisspezifische Pläne zu den folgenden Schadenslagen (gemäß Empfehlung des BBK):

Bitte bewerten Sie die Antwortmöglichkeiten

	Ja	Nein	Unbekannt
Evakuierung/Räumung des Krankenhauses	○	○	○
Brand im Krankenhaus	○	○	○
Massenanfall von Verletzten (MANV)	○	○	○
**CBRN**			
Biologische Gefahrenlage	○	○	○
Chemische Gefahrenlage	○	○	○
Radio-nukleare Gefahrenlage	○	○	○
**Polizeiliche Lagen**			
Amoklauf/Terroranschlag	○	○	○
Bombendrohung	○	○	○
Geiselnahme	○	○	○
**Naturgefahren**			
Hochwasser	○	○	○
Starkregen	○	○	○
**Störung kritischer Infrastruktur (KRITIS)**			
Ausfall der IT	○	○	○
Ausfall der elektrischen Energieversorgung	○	○	○
Ausfall der Wasserversorgung	○	○	○
Ausfall der Wärmeversorgung	○	○	○
Ausfall der Kommunikation	○	○	○

6 Personelle Maßnahmen

Es existiert eine ärztlich besetzte Stabsstelle Krisen-/Katastrophenmanagement

Bitte wählen Sie eine Antwortmöglichkeit

- ○ ja (MIT Freistellung)
- ○ ja (OHNE Freistellung)
- ○ nein
- ○ unbekannt

Im Krankenhaus kommt ein Sicherheitsdienst zum Einsatz

Bitte bewerten Sie die Aussage

- ○ ja
- ○ nein
- ○ unbekannt

7 Personelle Maßnahmen 2

Der Sicherheistdienst wird gestellt von:

Bitte wählen Sie eine Antwortoption

- ○ Klinikeigene Mitarbeiter
- ○ Externe Sicherheitsfirma
- ○ Beides

Haben Sie sonstige Kommentare zum Thema Sicherheit in Krankenhäusern?

Bitte geben Sie Anmerkungen/Kommentare hier ein (es dürfen KEINE personenbezogenen Daten eingetragen werden):

8 Endseite

Vielen Dank für Ihre Teilnahme!
